# The Dilemma of Heterogeneity Tests in Meta-Analysis: A Challenge from a Simulation Study

**DOI:** 10.1371/journal.pone.0127538

**Published:** 2015-05-29

**Authors:** Shi-jun Li, Hua Jiang, Hao Yang, Wei Chen, Jin Peng, Ming-wei Sun, Charles Damien Lu, Xi Peng, Jun Zeng

**Affiliations:** 1 Department of Computational Mathematics and Bio-statistics, Metabolomics and Multidisciplinary Laboratory for Trauma Research, Sichuan Provincial People’s Hospital, Sichuan Academy of Medical Sciences, Chengdu, Sichuan, China; 2 Department of Parenteral and Enteral Nutrition, Peking Union Medical College Hospital, Beijing, China; 3 Institute of Burn Research, Southwest Hospital of the Third Military Medical University, Chongqing, China; Shanghai Jiao Tong University School of Medicine, CHINA

## Abstract

**Introduction:**

After several decades’ development, meta-analysis has become the pillar of evidence-based medicine. However, heterogeneity is still the threat to the validity and quality of such studies. Currently, Q and its descendant I^2^ (I square) tests are widely used as the tools for heterogeneity evaluation. The core mission of this kind of test is to identify data sets from similar populations and exclude those are from different populations. Although Q and I^2^ are used as the default tool for heterogeneity testing, the work we present here demonstrates that the robustness of these two tools is questionable.

**Methods and Findings:**

We simulated a strictly normalized population S. The simulation successfully represents randomized control trial data sets, which fits perfectly with the theoretical distribution (experimental group: p = 0.37, control group: p = 0.88). And we randomly generate research samples Si that fits the population with tiny distributions. In short, these data sets are perfect and can be seen as completely homogeneous data from the exactly same population. If Q and I^2^ are truly robust tools, the Q and I^2^ testing results on our simulated data sets should not be positive. We then synthesized these trials by using fixed model. Pooled results indicated that the mean difference (MD) corresponds highly with the true values, and the 95% confidence interval (CI) is narrow. But, when the number of trials and sample size of trials enrolled in the meta-analysis are substantially increased; the Q and I^2^ values also increase steadily. This result indicates that I^2^ and Q are only suitable for testing heterogeneity amongst small sample size trials, and are not adoptable when the sample sizes and the number of trials increase substantially.

**Conclusions:**

Every day, meta-analysis studies which contain flawed data analysis are emerging and passed on to clinical practitioners as “updated evidence”. Using this kind of evidence that contain heterogeneous data sets leads to wrong conclusion, makes chaos in clinical practice and weakens the foundation of evidence-based medicine. We suggest more strict applications of meta-analysis: it should only be applied to those synthesized trials with small sample sizes. We call upon that the tools of evidence-based medicine should keep up-to-dated with the cutting-edge technologies in data science. Clinical research data should be made available publicly when there is any relevant article published so the research community could conduct in-depth data mining, which is a better alternative for meta-analysis in many instances.

## Introduction

Currently, Q and its descendent I^2^ tests are widely used, especially the I^2^ test, in meta-analysis [1–3]. Established in 2003 by Higgins et al, it is becoming the mainstay for testing heterogeneity [1]. Q and I^2^ tests have been integrated into *Review Manager* and almost all other meta-analysis software, and are used as the default tool to determine heterogeneity. In the past decade, along with the emergence of meta-analysis as a core technique for evidence-based approach in almost all branches of bio-medical research, Q and I^2^ make up an important methodological component of the enormous number of systematic reviews and clinical guidelines.

Unfortunately, despite the wide use and acceptance of Q and I^2^ tests, the work we present here demonstrates that the robustness of these two tools are questionable; and in many circumstances, relying solely on these tools to measure heterogeneity could lead to the wrong conclusion in meta-analysis, which forms the foundation of evidence-based medicine.

## Materials and Methods

### Theoretical Analysis and Simulation

#### Analyzing on the Structure of Q and I2

The structure of the equation of Q is the following:

$$\text{Q}=\sum_{k}{\hat{\omega }}_{k}{\left(\mu_{k}-{\hat{\bar{\mu }}}_{\hat{\omega }}\right)}^{2}$$ (1)

$${\hat{\omega }}_{k}=n_{k}/\sigma_{k}^{2}$$ (2)

Here, ${\bar{\mu }}_{\hat{\omega }}=\sum \omega_{k}\mu_{k}/\sum_{k}\omega_{k}$ and represents the weight of the *k*-th study, *n*_*k*_ is the sample size of the *k*–th study. It is assumed that the sample from any trial is independent and the distribution is normalized [3].

Q does not consider the influence from the number of enrolled trials (degree of freedom, *df)*. We can understand this shortcoming of Q from its equation: Q is the weighted sum of the squares of deviations (WSSD) of data sets from the enrolled trials. Along with the increase of the number of trials (n), the non-negative term also increases. Therefore, the number of enrolled trials significantly influences the increase of Q value. Thus the increase of Q value cannot simply be attributed to the variants between enrolled trials. To overcome this shortcoming, Higgins *et al* constructed I^2^. It modifies Q and aims to balance the extra variant, which comes from the increase of the number of enrolled trials. Strictly speaking, I^2^ is not a test but a descriptive measure.

The equation of I^2^ is the following

$$I^{2}\;=\frac{Q-df}{Q}\times 100\%$$ (3)

Here *df* is the degree of freedom, *df* = n-1

Although I^2^ proposes to overcome quasi-heterogeneity from extra variants, a more serious influence is not considered, which is the sample size n_k_ (Eq.2). We can easily find that ${\hat{\omega }}_{k}$ is in proportion to *n*_*k*_. Along with the increase of the sample size, the corresponding deviation will also increase. Consequently, the Q value will increase.

Let

$$\text{T}=\frac{\mu_{k}-{\hat{\bar{\mu }}}_{\hat{\omega }}}{\frac{\sigma_{k}}{\sqrt{n_{k}}}}$$ (4)

Remember that the default assumption behind the statistics of the t-test is that the distribution of all enrolled trials met $\text{N}\;\left(\mu_{k},\sigma_{k}^{2}\right)$ and we therefore have T ∼ T (*n*_*k*_ − 1) → *N* (0, 1). So Q is indeed the sum of the squares of T_k_. Consequently, constructing Q is a process that is made up by the sum of the square of T_k_. It is easy to infer that the sample size of each trial cannot be too big, otherwise the T value will surge.

To explore the evolutionary patterns between Q, I^2^ and n_k_, we herein introduce a simulation process to verify the influence of N and n to Q and I^2^.

#### Simulation Process

**Fig 1:**
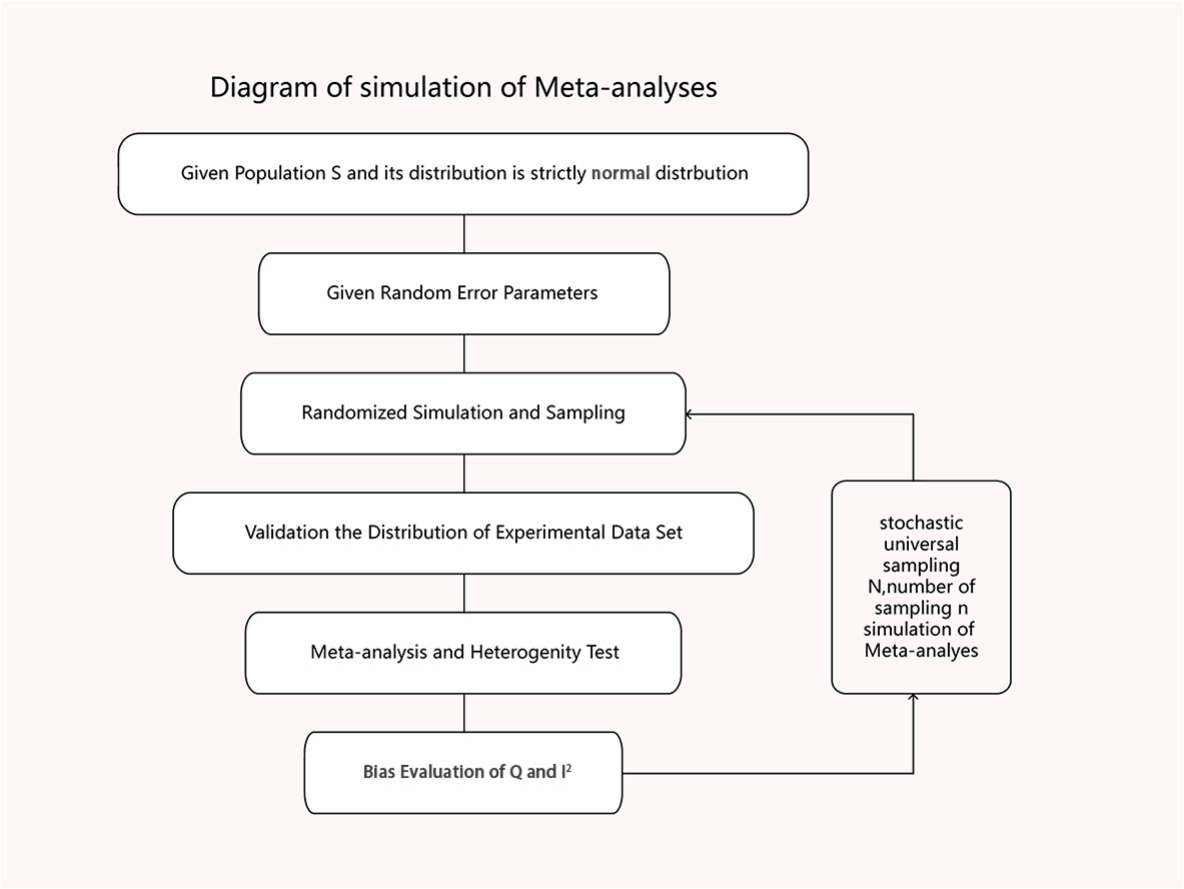
Diagram of the simulation process.

We illustrated the research flow and simulation process of the study in Fig 1.

We simulated a population S and its distribution is strictly normalized, which means S∽*N* (μ, σ^2^) (Table A in S1 File). Now we have samples S_i_ (i = 1, 2, 3…n) where each is a random sample from S (Table B in S1 File). Let S_i_∽(μ_i_, σ_i_^2^). The variation between the samples is only made by random error ε, and ε∽*N*(0,σ_ε_).

The distribution parameters of Si can be descripted as following :

$$\mu_{i}=\mu +E\left(\varepsilon \right)$$ (5)

$$\sigma_{i}=\sqrt{\sigma^{2}+\sigma_{\varepsilon }^{2}}$$ (6)

Let σ_ε_<<σ, then we have:

$$\text{E}\left(S_{i}\right)=\mu_{i}=\mu +0=\mu =E\left(S\right)$$ (7)

$$\text{D}\left(S_{i}\right)=\sigma_{i}^{2}=\sqrt{\sigma^{2}+\sigma_{\varepsilon }^{2}}\approx D\left(\text{S}\right)$$ (8)

We know Si is a non-skewed sampling of the population. Therefore the simulated data sets are homogenous. We then synthesized these data sets by meta-analysis (fixed model, meta: meta-analysis with R was employed for data aggregating) and we calculated Q and I^2^ for each synthesis experiment (Tables C and D in S1 File). To each Si, sampling process will be repeated in 1000 times. Thus we get the distribution of I^2^ variations in synthesizing different number of trials (the sample size of each trial is the same). Finally we generated heat map to see the impact of I^2^, Q and the number of trials (n) and sample size N (Tables E, F and G in S1 File)

#### Distribution Test

We used Kolmogorov-Smirnov Tests to test the distribution of the samples,α = 0.05.

#### Simulation Algorithm

We employed Mersenne-Twister (Matsumoto and Nishimura, 1998) from RNG to simulate data sets [4, 5]. Simulation programming in R see Tables A-G in S1 File.

#### Environment and Setting of Computation

All computing processes were done using a high performance-computing platform at the Sichuan Academy of Medical Sciences, by using R (version 3.1.1 for win7 64bit) [4].

## Results and Discussion

**Fig 2:**
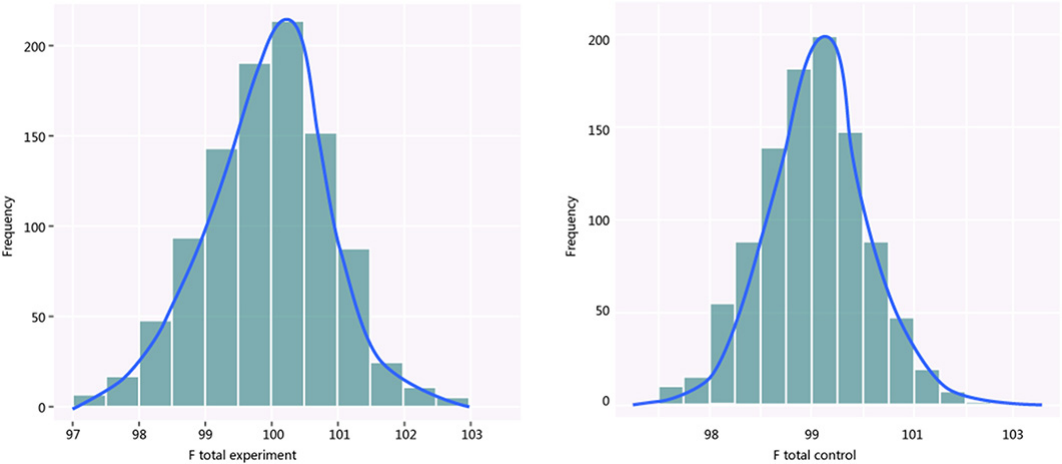
Distribution of simulated S, which is typical normal distribution. (A: Experimental group; B: control group).

**Fig 3:**
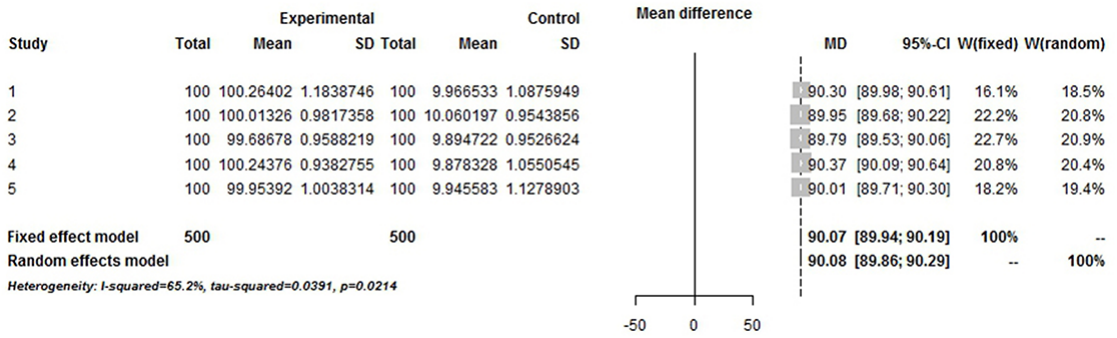
Simulated Meta-analysis. Enrolled 5 trials, total number 1000, pooled MD 90, I^2^ = 65.2%.

**Fig 4:**
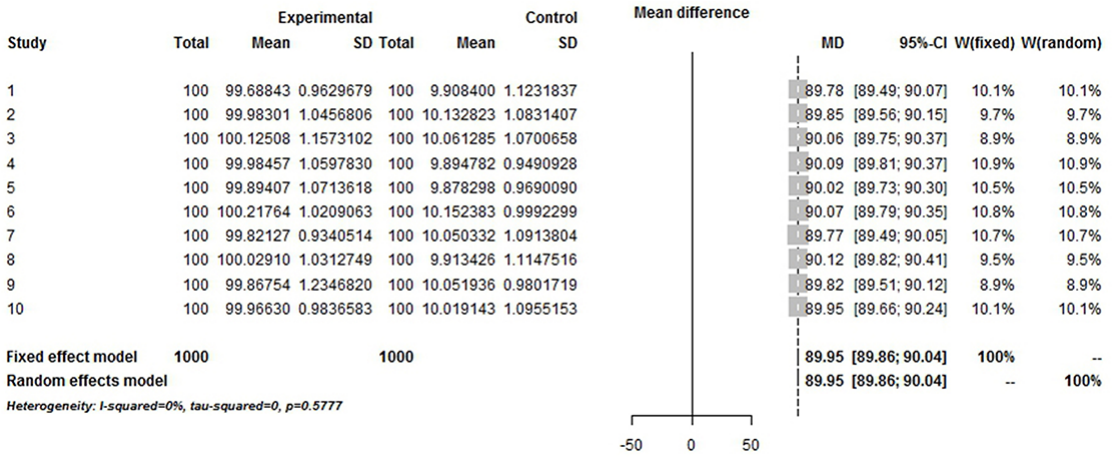
Simulated Meta-analysis. Enrolled 10 trials, total number 2000, pooled MD 89.95, I^2^ = 0%.

**Fig 5:**
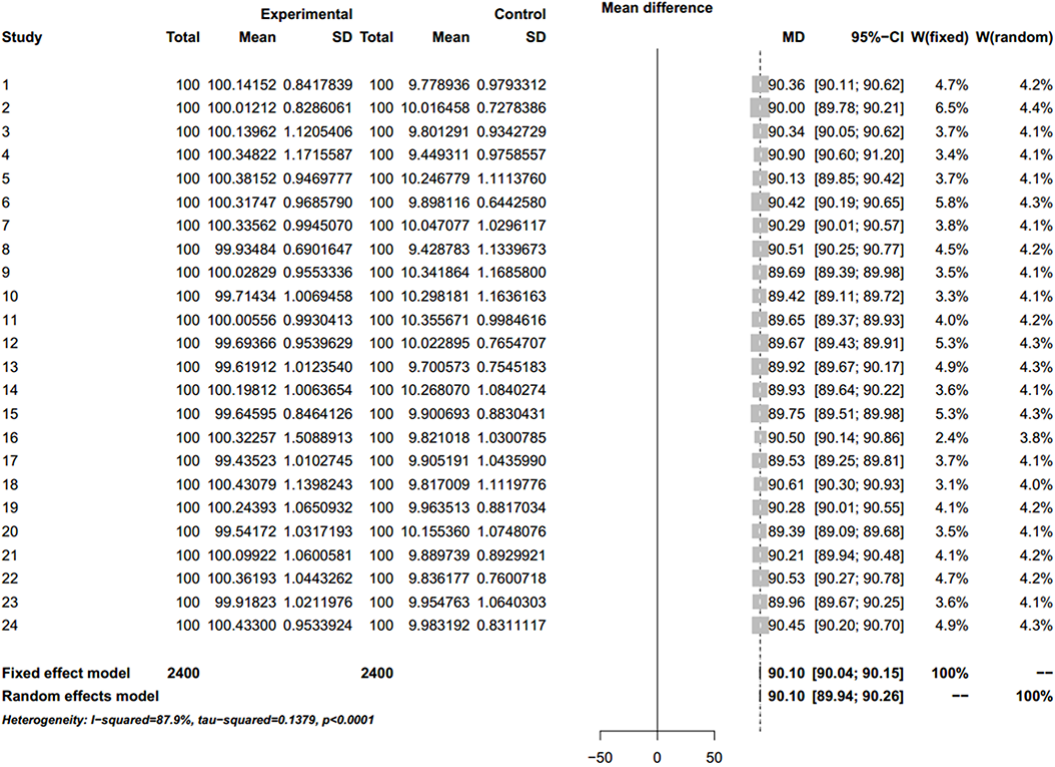
Simulated Meta-analysis. Enrolled 24 trials, total number 2400, pooled MD 90.1, I^2^ = 87.9%.

**Fig 6:**
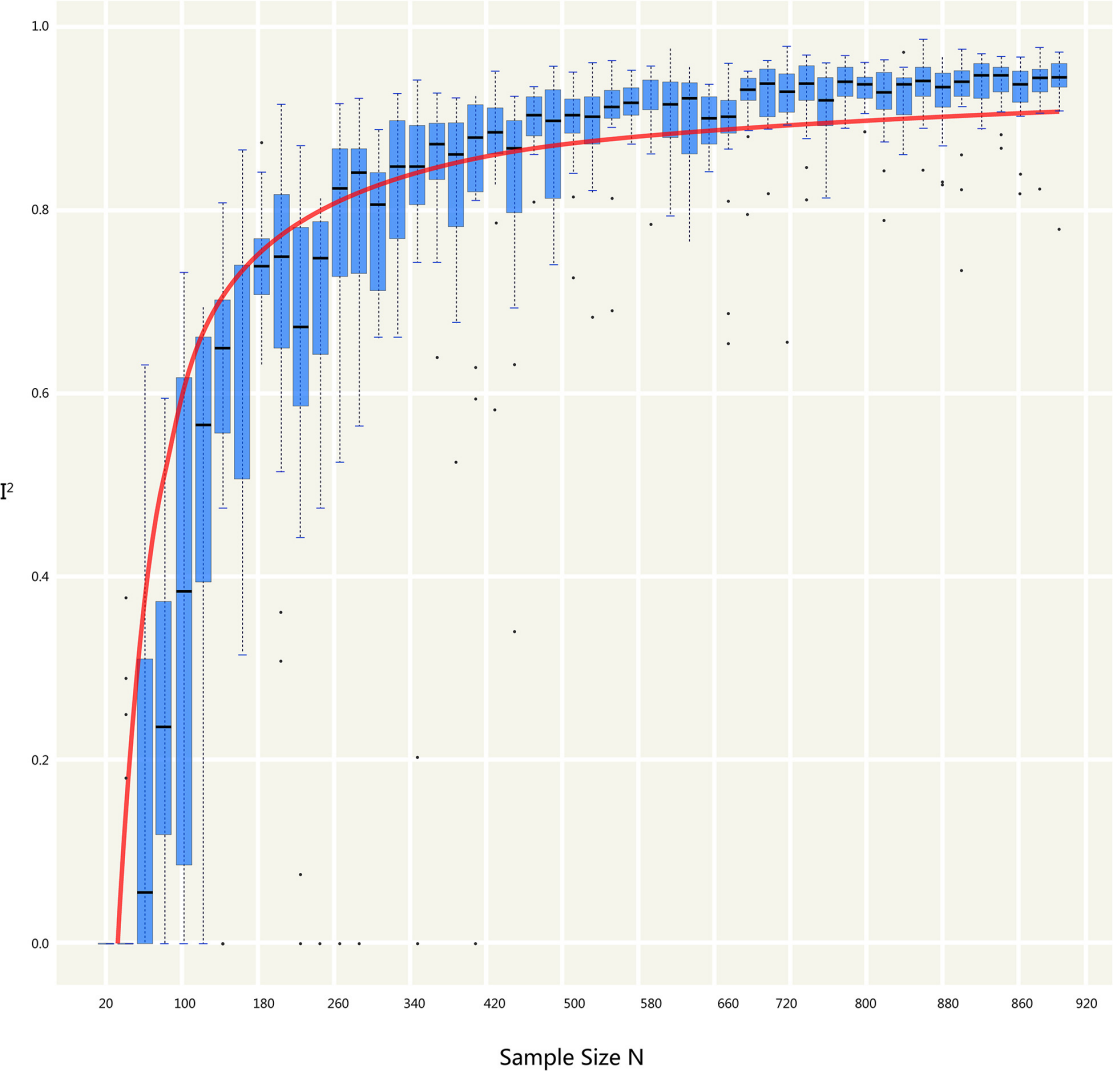
Impact of I^2^ and the sample size. Lateral axis represents the sample size; vertical axis represents the I^2^ value. Boxes represent the distribution of I^2^ variations in synthesizing different number of trials (the sample size of each trial is the same). To each Si, sampling process will be repeated in 1000 times.

**Fig 7:**
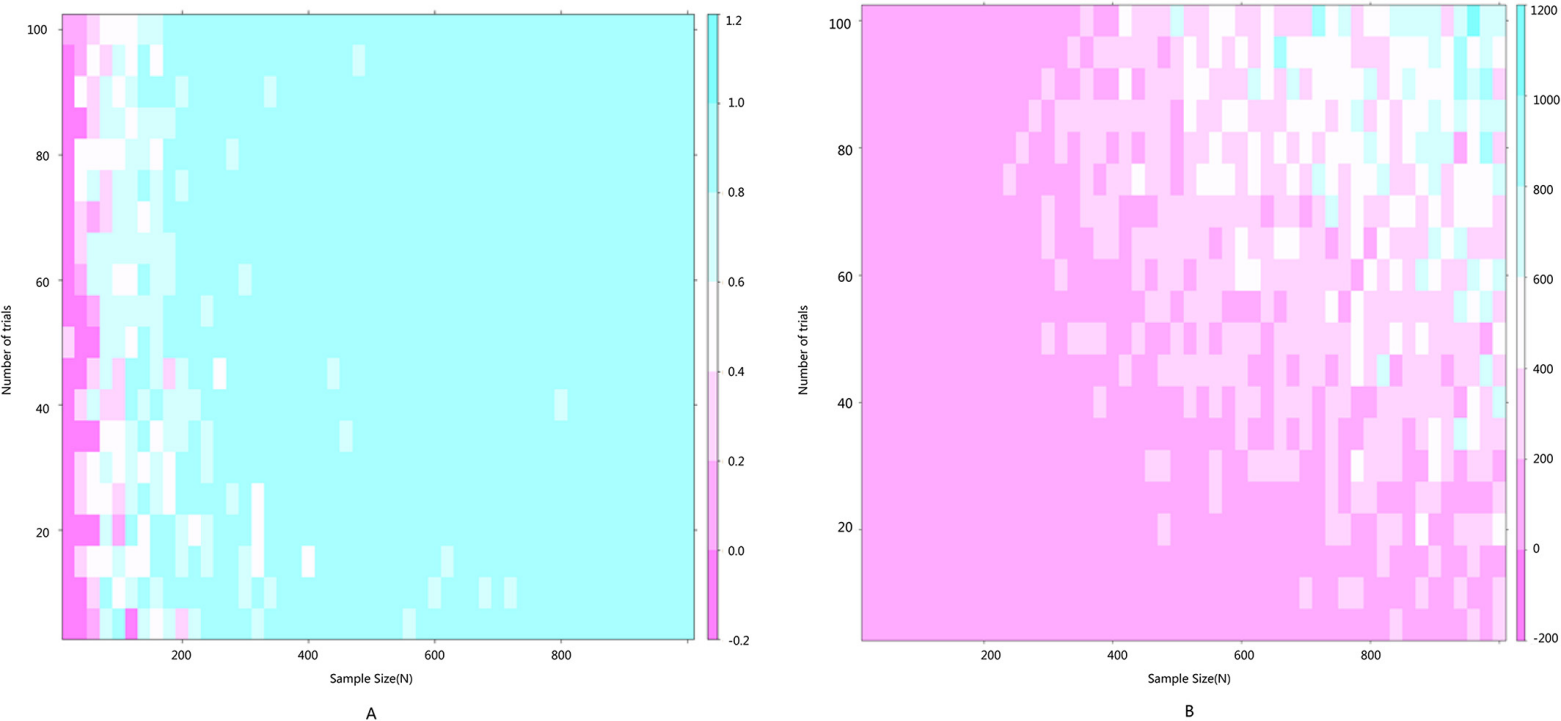
Heat maps of the impact of two heterogeneity test tools. A: Heat map of I^2^ and the number of trials (n) and sample size N; B: Heat map of Q and the number of trials (n) and sample size N;.

The simulation successfully represents randomized control trial data sets that meet normal distribution and generates *S* (Table 1 and Fig 2), which fits perfectly with the theoretical distribution (experimental group: p = 0.37, control group: p = 0.88). And we randomly generate research samples S_i_ that fits the population with tiny distributions. In short, these data sets are perfect and can be seen as completely homogenous data from the exactly same population. If Q and I^2^ are truly robust tools, the Q and I^2^ test results on our simulated data sets here should not be positive. We then synthesized these trials by using fixed model. We exhibit here three meta-analyses that are selected from our simulation experiments (Figs 3–5). Pooled results indicated that the mean difference (MD) corresponds highly with the true values, and the 95% is narrow. But, along with the increase of the numbers of trials and sample size, the value of the I^2^ steadily increased (Figs 6 and 7A). Relatively, the influence of number of trials is relatively smaller. In terms of Q, we found that the value of Q increases along with the increase of the number of trials synthesized into the meta-analysis, and with the increase of the sample sizes of enrolled trials (Fig 7B).

**Table 1:** Distribution of simulated data sets.

Parameters of Distribution	True value of S (population)	Estimation of simulated S	Error	P
Experimental Group				0.37
μe	100	99.99	0.01	
σe	1	0.963	0.037	
Control group				0.88
μc	10	10.01	0.01	
σc	1	1.059	0.059	

### Forest plots of Simulated Meta-analysis

We demonstrate here that the validity of Q and I^2^ test is questionable and unstable to evaluate heterogeneity for meta-analysis. The purpose of the heterogeneity test is to determine whether the included trials are sampled from similar populations. If the samples of included trials are from similar populations, then the expected mean of the samples should equal the mean of the populations (true data). If it is not, then the mean of the samples does not equal the mean of the populations (false data). The core philosophy of meta-analysis is to include those trials from populations that are *de facto* the same. The mission of any heterogeneity test is to detect the trials that are *de facto* not the same. A good heterogeneity-testing tool therefore should not make the mistake to classify a homogenous trial as heterogeneous.

Because all the data sets of the simulated enrolled trials in our study are from the sample population, there could be no heterogeneity between them. When the sample size is small, the bias from sampling will increase with the frequency of sampling. When sampling increases in frequency, the theoretical true bias will decrease, thus heterogeneity should decrease. The mean and variance tend to stabilize when the sampling frequency continues to increase. In this scenario, the I^2^ and Q value will increase proportionally along with the sample size n_k_, thus causing the quasi-heterogeneity. In summary, both Q and I^2^ are sensitive and dependent on sample size n_k_ (Fig 6 and Fig 7).

Gerta Rücker et al have published an article in 2008 also tried to address the I^2^ problem [6]. The result of Rücker’s study seemed similar to ours: we both reached the conclusion that the I^2^ will increase to 100% along with the sample size increasing in a meta-analysis. But, there was a major methodological flaw in Rücker’s study, which it was the fact that they did not test the homogeneity and distribution of the data sets included in their simulation. As is well known, most people performing meta-analysis do not conduct distribution tests on their data set from the original trials, and heterogeneity is quite real in most circumstances. Because the data sets of Rücker’s study are from real meta-analysis which quite possibly contains high heterogeneous trials, it is impossible to get rid of the heterogeneity risk by directly and randomly sampling from these data sets. In other words, when the sample size is large enough and the heterogeneity is de facto existent, the increase of I^2^ is most likely expected. But, such a simulation cannot be seen as a strict mathematic proof. What we did in our study was to give the complete proof in full generality. In short, we simulated a pure homogenous population S and strictly normalized its distribution, and then we repeated the sampling in 1000 times and proved the I^2^ was unstable in any case when sample size increased. To our best knowledge, the study we presented here is the very first one that generally proved that using I^2^ test can lead erroneous results in any case when sample size of a meta-analysis is large.

After several decades’ development, meta-analysis has become a pillar of evidence-based medicine. But heterogeneity is still the threat to the validity and quality of meta-analysis. The core issue is to distinguish data sets from similar populations and exclude the others. First of all, currently meta-analysis researchers accept the data expressed as mean±sd by default as normal distribution, without any further analysis to test whether this distribution hypothesis is correct or not. Thus the heterogeneity challenge is quite real.

Secondly, almost none of the clinical researchers are aware that Q and I^2^ are tools that can only be applicable to test heterogeneity between small sample size trials, and will lost their robustness when the sample sizes and the number of trials are substantially increased (as demonstrated by our study presented here).

This represents a dilemma: the purpose of meta-analysis is to enlarge the sample size, in order to expand and validate the implication of the result. New meta-analysis researches including these flaws are emerging and passed on to clinical practitioners as “updated evidence”, but they are actually not strong as they assumed.

## Conclusions

In summary, the validity of widely used Q and I^2^ test in current meta-analysis is questionable and unstable on heterogeneity evaluation. Before new heterogeneity evaluation tool which is developed and its robustness are demonstrated, we will suggest more strict applications of meta-analysis. The meta-analysis may only be applied to those synthesized trials with small sample sizes. We call upon that the tools of evidence-based medicine should keep up-to-dated with the cutting-edge technologies in data science. Clinical research data should be made available publically when there is any relevant article published so the research community could conduct in-depth data mining, which is a better alternative for meta-analysis in many instances.

## Supporting Information

S1 File Tables A to G. Code of simulation algorithm and graphics plot in R.
(DOCX)
