# Supplementary material for: The Dilemma of Heterogeneity Tests in Meta-Analysis: A Challenge from a Simulation Study
Source: PLoS One. 2015 May 29;10(5):e0127538. doi: 10.1371/journal.pone.0127538 (PMC4449216; doi:10.1371/journal.pone.0127538)
Supplement: S1 File — Code of simulation algorithm and graphics plot in R. (DOCX) [file pone.0127538.s001.docx]

*PLOS ONE* Supporting Information (SI)

Code of simulation algorithm and graphics plot in R

Table A：

| **Algorithm 1** Simulated S |
| --- |
| **Initialization：**  Set U.total.experiment=100; D.total.experiment=1; U.total.control=10; D.total.control=1; N.total=10000; |
| **Iteration:**  1: F.total.experiment = rnorm(n = N.total, mean = U.total.experiment, sd = D.total.experiment);  2: F.total.control = rnorm(n = N.total, mean = U.total.control, sd = D.total.control);  **Output：**  Generate Population S; |

Table B：

| **Algorithm 2** Simulated S_i_ |
| --- |
| **Initialization：**  Set U.total.experiment=100; D.total.experiment=1; U.total.control=10; D.total.control=1; U.error =0；D.error=0.1；N.sample={10,15,20,…,100}; n.samples={4,5,6,…24}; |
| **Iteration:**  1: F.sample.all.experiment = list();  2: F.sample.all.control = list();  3: F.total.experiment = rnorm(n = N.total, mean = U.total.experiment, sd = D.total.experiment)；  4: F.total.control = rnorm(n = N.total, mean = U.total.control, sd = D.total.control);  5: ram.seed = rnorm(n.samples,U.error,D.error);  6: F.sample.all.experiment=  lapply(ram.seed,ramdomfunc,mean.ram=U.total.experiment,sd.ram=  sqrt((D.total.experiment^2+D.error^2)));  7: ram.seed = rnorm(n.samples,U.error,D.error);  8: F.sample.all.control =  lapply(ram.seed,ramdomfunc,mean.ram=U.total.control,sd.ram=  sqrt((D.total.control^2+D.error^2)));  9: R.all.mean.experiment = sapply(F.sample.all.experiment,mean);  10: R.all.sd.experiment = sapply(F.sample.all.experiment,sd);  11: R.all.mean.control = sapply(F.sample.all.control,mean);  12: R.all.sd.control = sapply(F.sample.all.control,sd) ;  **Output：**  Generate Population Si  Return R.all.mean.experiment, R.all.sd.experiment, R.all.mean.control, R.all.sd.control; |

Table C: meta-analysis：

| **Algorithm 3** meta-analysis |
| --- |
| **Initialization：**  Set R.all.mean.experiment; R.all.sd.experiment; R.all.mean.control; R.all.sd.control); |
| **Iteration:**  1: meta.data <-  data.frame(R.all.mean.experiment,R.all.sd.experiment,R.all.mean.control,R.all.sd.control)  2: meta.model.result <- metacont(n.e =  rep(N.sample,n.samples),mean.e = R.all.mean.experiment,sd.e = R.all.sd.experiment,  n.c = rep(N.sample,n.samples),mean.c = R.all.mean.control,sd.c = R.all.sd.control)  **Output：**  calculate meta-analysis ,  return Q, I2, n.samples, N.sample; |

Table-D:I^2^：

| **Algorithm 4** I2(n,N) boxplot |
| --- |
| **Initialization：**  Set I2.list, n.samples, N.sample; |
| **Iteration:**  1: I2.data.frame = as.data.frame(I2.list);  2： I2.matrix = as.matrix(I2.data.frame);  3: I2.matrix.t = t(I2.matrix)  4: boxplot(I2.matrix.t);  **Output：**  Hetamap for I2-n,N; |

Table E:I^2^ Thermal graphs：

| **Algorithm 5** I2(n,N) heatmap |
| --- |
| **Initialization：**  Set I2.list, n.samples, N.sample; |
| **Iteration:**  1: I2.data.frame = as.data.frame(I2.list);  2： I2.matrix = as.matrix(I2.data.frame);  3: I.x =s eq(from=1,to=n.samples,by=1);  4: I.y = seq(from=1,to=N.sample,by=5);  5: grid = expand.grid(x=I.x, y=I.y);  6: grid$I2 = as.numeric(unlist(I2.list));  7: contourplot(I2~x*y,grid,pretty=T,region=T,contour=F,xlab="sample size",ylab="number of trials ")  **Output：**  Hetamap for I2-n,N; |

Table F: Q, n and N：

| **Algorithm 6** Q(n,N) boxplot |
| --- |
| **Initialization：**  Set Q.list, n.samples, N.sample; |
| **Iteration:**  1: Q.data.frame = as.data.frame(I2.list);  2： Q.matrix = as.matrix(I2.data.frame);  3: Q.matrix.t = t(I2.matrix)  4: boxplot(Q.matrix.t);  **Output：**  Hetamap for Q-n,N; |

Table G: Q Thermal graph:

| **Algorithm 7** I2(n,N) heatmap |
| --- |
| **Initialization：**  Set Q.list, n.samples, N.sample; |
| **Iteration:**  1: Q.data.frame = as.data.frame(I2.list);  2： Q.matrix = as.matrix(I2.data.frame);  3: Q.x =s eq(from=1,to=n.samples,by=1);  4: Q.y = seq(from=1,to=N.sample,by=5);  5: grid = expand.grid(x=Q.x, y=Q.y);  6: grid$Q = as.numeric(unlist(Q.list));  7: contourplot(Q~x*y,grid,pretty=T,region=T,contour=F,xlab="sample size",ylab="number of trials ")  **Output：**  Hetamap for Q-n,N; |
